# Lower breast cancer survival in mothers of children with a malignancy: a national study

**DOI:** 10.1038/sj.bjc.6604350

**Published:** 2008-05-06

**Authors:** H Olsson, S Magnusson, A Bladström

**Affiliations:** 1 Department of Oncology, Institution of Clinical Sciences, Lund University, Lund SE-221 85, Sweden; 2 Department of Cancer Epidemiology, Institution of Clinical Sciences, Lund University Hospital, Lund SE-221 85, Sweden

**Keywords:** breast cancer, childhood cancer, familial cancer, hereditary syndrome, prognosis

## Abstract

As it is unclear if hereditary factors affect breast cancer survival, this was compared using fertility and cancer registry data, among all women so diagnosed during 1961–1999 in Sweden, having a child with childhood cancer (⩽20 years of age; *n*=254) and with that of other women (*n*=74 781). Those having a child with a childhood malignancy had a significantly worse survival than other women, relative risk (RR)=1.25, 95% CI 1.02–1.55, *P*<0.04, adjusted for age at diagnosis, year of diagnosis, parity and time since last pregnancy. Childhood sarcomas or acute myeloid leukaemia seemed to be most associated with a worse survival in the mother (RR=1.38 and 1.69, respectively). The lower survival of the mother was present for breast cancer diagnosed both before and after 50 years of age. The Li–Fraumeni syndrome and possibly other genetic disorders may lower breast cancer survival.

Only a minority of cases of breast cancer have a hereditary background. Hereditary factors could affect tumour biology and disease prognosis and it has been suggested that breast cancer patients having p53 germline mutations have an especially bad prognosis (Olsson, 2000, 2001). In the present study, survival has been compared in women with breast cancer with and without having a child with a paediatric tumour, using the latter as a surrogate measure for such hereditary factors as Li–Fraumeni syndrome (Li and Fraumeni, 1969; Malkin *et al*, 1990; Varley *et al*, 1997).

## MATERIALS AND METHODS

Women with at least one child and a breast cancer diagnosis during 1961–1999 were identified by means of the unique personal identification numbers and the National Swedish Cancer register, fertility register and Swedish national censuses (*n*=75 035) together with deaths, emigrations and tumours in their children. Only primary tumours were considered and no information about recurrences or development of secondary tumours is available. The women were followed from breast cancer diagnosis to the first event of emigration, death or 31 December 2001.

In addition, deaths after breast cancer in women having a child with any of the diagnosis of sarcoma, brain tumour, lymphoma, acute leukaemia or myeloid leukaemia ⩽20 years of age, compared to women not having any child with cancer were analysed by means of Cox proportional hazard modelling. We also adjusted for the number of children and time since last childbirth, which are known to affect prognosis. However, results by childhood tumour type should be interpreted with caution, as there were only few women in each relevant group. All analyses were stratified for the women's year and age of diagnosis.

Kaplan–Meier survival curves for women having a child with any of the specified diagnoses and women not having any child with cancer were also estimated. To have groups with a comparable year and age of diagnosis, four women who did not have any child with cancer matched for year and age of diagnosis were randomly selected for each woman who had a child with cancer. Kaplan–Meier survival curves were also estimated for women with a diagnosis at <50 and ⩾50 years of age, respectively.

The Research Ethics Committee of Lund University approved this study.

## RESULTS

A total of 254 women with breast cancer having a child with malignancy were identified, of which 33 were diagnosed before the childhood tumour. Brain tumours, leukaemia, lymphomas and sarcoma were the commonest childhood tumours.

As shown in Table 1, the relative risk (RR) of dying for a woman with breast cancer having a child with cancer *vs* not having a child with cancer is 1.26 (95% CI 1.02–1.56). Risk was highest if the child had acute myeloid leukaemia (RR=1.71, 95% CI 0.75–3.89) or sarcoma (RR=1.42, 95% CI 0.88–2.28). Adjusting for number of children and time since last childbirth only marginally changed the risk estimates. Censoring the follow-up of all women at 65 years of age did not influence the results.

**Figure 1:**
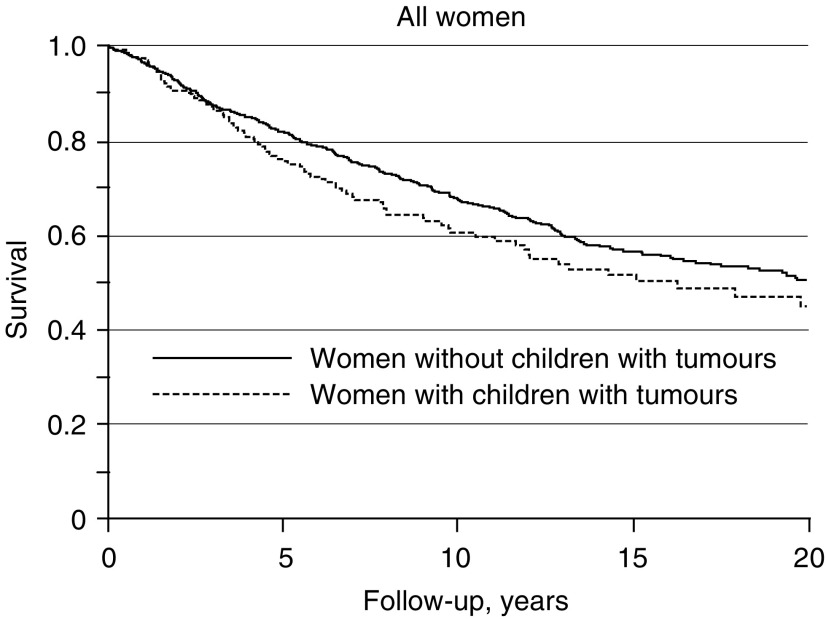
Kaplan–Meier survival curves for women with and without a child with a malignant tumour given for all women.

Figure 1 shows the survival curve for all women. The survival difference was present both in younger (<50 years) and older (⩾50 years at diagnosis) patients and seemed to persist beyond 15 years of follow-up. However, the difference occurred earlier and was more pronounced in younger patients.

## DISCUSSION

In this population-based national registry study, a worse breast cancer survival was found for women also having an offspring with a paediatric malignancy compared to other parous women not having a child with malignancy. The most plausible explanation is that a genetic factor, such as p53 germline mutations, in a small subgroup of the first-mentioned women, also affects tumour biology causing the worse survival, as we postulated before (Olsson, 2001). Breast cancer is the commonest tumour disease in the Li–Fraumeni syndrome, cumulatively with about a 50% lifetime risk in female carriers (Eeles *et al*, 1993; Chompret *et al*, 2000; Varley, 2003). The childhood tumours such as sarcoma, brain tumours and leukaemia described here are well-recognised within the syndrome (Chompret *et al*, 2000).

In this registry study, we lacked information about tumour stage or other biological factors related to prognosis and about therapy. We were, however, able to adjust for age at diagnosis, birth year, number of children and time since last childbirth, as breast cancer diagnosed in pregnancy or in its close proximity has an especially poor prognosis (Guinee *et al*, 1994; Bladstrom *et al*, 2003); this only marginally changed the risk estimates. It is, however, unlikely that access to different therapy or a biological factor randomly could account for the findings.

Being a nationwide study, overall survival was estimated and not breast cancer-specific survival. Intercurrent disease and death could have affected survival in women with diagnosis at older ages (analysed above 60 years of age) but would have no major effect in younger women. Thus, the smaller effect seen for older patients could be due to differences in intercurrent deaths.

There is no reason to suspect inclusion bias that an offspring having childhood cancer is related to the likelihood of a woman being identified by the registries. Most childhood cancers occurred before the breast cancer diagnoses in mothers, but available evidence gives little support to the notion that a stress reaction or grief can affect the prognosis of breast cancer. However, an alternative hypothesis is that the shared environmental effects might also contribute to an increased mortality risk.

Further studies with tumour stage, hormone receptor status, nuclear grade, erbb2 status, therapy and p53 germline mutation status may elucidate this link between childhood cancer and breast cancer. Our results suggest that data on whether a woman has had an offspring with childhood cancer have prognostic value.

## Figures and Tables

**Table 1:** Relative risk of death in women with breast cancer not having a child with malignancy and in women with breast cancer having a child with a malignancy according to childhood tumour type

**Group**	**Number of women^a^**	**Unadjusted RR (95% CI) *P*-value**	**Adjusted^b^ RR (95% CI) *P*-value**
Child without cancer	74 781	1	1
Child with cancer	254	1.26 (1.02–1.56)	1.25 (1.02–1.55)
		0.03	0.04
			
*By specified tumour type*			
Sarcoma	43	1.42 (0.88–2.28)	1.38 (0.86–2.22)
		0.15	0.18
Brain tumour	81	1.19 (0.80–1.76)	1.18 (0.80–1.75)
		0.39	0.40
Lymphoma	58	1.31 (0.86–1.99)	1.28 (0.85–1.96)
		0.21	0.24
Acute lymphatic leukaemia	34	0.88 (0.44–1.73)	0.89 (0.44–1.81)
		0.71	0.75
Myeloid leukaemia	20	1.71 (0.75–3.89)	1.69 (0.74–3.85)
		0.20	0.21
Monocytic leukaemia and other unspecified leukaemias	21	1.29 (0.66–2.50)	1.30 (0.67–2.54)
		0.46	0.43

RR=relative risk.

a One woman had a child with lymphoma and one child with acute lymphatic leukaemia; and one woman had one child with sarcoma, one child with brain tumour and one child with lymphoma; 33 women had the breast cancer diagnosis before the diagnosis of the child; and 221 women had the breast cancer diagnosis after the diagnosis of the child.

b Adjusted for number of children and time since last childbirth.
